# Podocytes in health and glomerular disease

**DOI:** 10.3389/fcell.2025.1564847

**Published:** 2025-04-24

**Authors:** Joanna Cunanan, Daniel Zhang, Anna Julie Peired, Moumita Barua

**Affiliations:** ^1^ Division of Nephrology, Toronto General Hospital, University Health Network, Toronto, ON, Canada; ^2^ Toronto General Hospital Research Institute, University Health Network, Toronto, ON, Canada; ^3^ Institute of Medical Sciences, University of Toronto, Toronto, ON, Canada; ^4^ Department of Experimental and Clinical Biomedical Sciences “Mario Serio”, University of Florence (Università degli Studi di Firenze), Florence, Italy

**Keywords:** podocytes, glomerular disease, actin cytoskeleton, slit diaphragm, differentiated, metabolism, therapeutic targets

## Abstract

Podocytes are highly specialized, terminally differentiated cells in the glomerulus of the kidney and these cells play a central role in blood filtration. In this review, we comprehensively describe the cell biology of podocytes under healthy conditions and in glomerular disorders wherein podocyte injury is a major pathological mechanism. First, the molecular mechanisms that maintain podocyte actin cytoskeleton structure, permanent cell cycle exit, and metabolism under healthy conditions are described. Secondly, the mechanisms of podocyte injury, including genetic alterations and external insults that ultimately disrupt podocyte actin cytoskeleton dynamics or interrupt podocyte quiescence and mitochondrial metabolism are discussed. This understanding forms the basis of described potential therapeutic agents that act by modulating dysregulated podocyte cytoskeleton organization, prevent or reverse cell cycle re-entry, and re-establish normal mitochondrial energy production. Lastly, the application of modern techniques such as single cell RNA sequencing, super resolution microscopy, atomic force microscopy, and glomerular organoids is improving the resolution of mechanistic podocytopathy knowledge. Taken together, our review provides critical insights into the cellular and molecular mechanisms leading to podocyte loss, necessary for the advancement of therapeutic development in glomerular diseases.

## 1 Introduction

Chronic kidney disease (CKD) is a major global health concern affecting 1 in 10 individuals, representing over 850 million people worldwide and resulting in more than 1 million deaths per year (Jha et al., 2013; Verberne et al., 2019; Jager et al., 2019). Care for patients with CKD presents a significant healthcare burden, with hospitalization costs of over 100 billion USD per year (Jha et al., 2013; Verberne et al., 2019; Jager et al., 2019).

**FIGURE 1:**
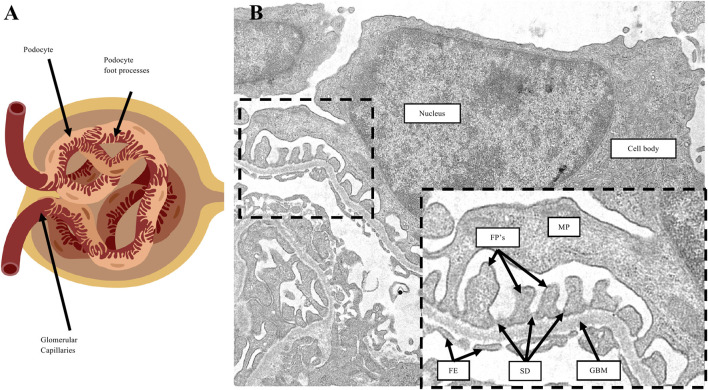
Podocyte morphology. **(A)** Diagram representation of a glomerulus. Podocytes are specialized epithelial cells comprised of cellular projections called foot processes which wrap around the surface of glomerular capillaries. **(B)** Transmission electron microscopy image of a podocyte. The cell body of podocytes branch into major processes (MP), which in turn form the foot processes (FP’s) that are in direct contact with the underlying glomerular basement membrane (GBM). On the capillary lumen side are fenestrated endothelium (FE). Adjacent foot processes form a space in-between called the slit diaphragm (SD).

Glomerular diseases are a major cause of CKD. Glomeruli (Figure 1) are the blood-filtering units of the kidney comprised of four specialized epithelial cells consisting of podocytes, endothelial cells, mesangial cells, and parietal epithelial cells. Podocytes (Figure 1) are highly specialized, terminally differentiated cells that serve a crucial role in glomerular filtration function. Podocyte loss defines many forms of glomerular disease, which are challenging to treat given the highly specialized, differentiated state of these cells. In this review, we focus on podocytes and the pathologic molecular mechanisms that define their role in disease. Ultimately this knowledge is critical to identify molecular targets and pathways for therapeutic advancement.

### 1.1 Glomerular diseases characterized by podocyte loss in kidney disease patients

Glomerular diseases represent approximately 85% of CKD in the USA and Western Europe (Grahammer and Huber, 2016). Disruption of the glomerular filtration barrier at the capillary wall leads to nephrotic syndrome characterized by >3 g/day proteinuria, hypoalbuminemia, hyperlipidemia, and edema (Ding and Saleem, 2012; Andolino and Reid-Adam, 2015). Broadly, there are 3 major histopathologic lesions observed in nephrotic syndrome which are descriptively labeled as minimal change disease, focal segmental glomerulosclerosis (FSGS) and membranous nephropathy (Vivarelli et al., 2017; Bose et al., 2014; Ronco et al., 2021). Podocyte abnormalities is a hallmark feature in minimal change disease and FSGS, manifested as disruption of the highly organized cell-cell junction known as the slit diaphragm, referred to as foot process effacement. However, podocyte injury–whether central or secondary as a result of the complex cellular interrelationships within the glomerulus–is a major contributor to the development and progression of glomerular diseases (Kriz, 2002). Podocyte damage, along with its potential for recovery or lack thereof, is a defining event in the chronic kidney disease pathway (Kriz, 2002). Given that podocytes have limited replicative capacity, the primary response to its loss is podocyte hypertrophy (Wiggins et al., 2005). When this compensatory response is exceeded (≥20% podocyte loss *in vivo*), then there is mechanical strain, detachment and exposure of the glomerular basement membrane precipitating a sequence of events culminating in glomerular scarring (Wharram et al., 2005; Kretzler et al., 1994; Kriz, 2003).

### 1.2 Experimental models of podocyte loss

Clinically relevant experimental models of podocytopathies have proven useful to delineate cellular and molecular mechanisms governing glomerular diseases. Among the most commonly employed rodent models are the use of podocytotoxic agents such as in adriamycin nephropathy (Lee and Harris, 2011), puromycin aminonucleoside (Marshall et al., 2006), long term stimulation by FGF-2 (Kriz et al., 1995), Masugi nephritis (induction of glomerulonephritis using anti-kidney serum) (Shirato et al., 1996), and Thy-1.1 glomerulonephritis (induction of mesangial proliferative glomerulonephritis using an anti-Thy1.1 monoclonal antibody) (Kriz et al., 2003); as well as different genetic models such as knockout or loss-of-function mutations in key podocyte proteins like nephrin, podocin, and podocalyxin (Verma et al., 2018; Refaeli et al., 2019; Fan et al., 2009; Gigante et al., 2009). *Drosophila* nephrocytes have also been used as podocyte models, particularly to study the glomerular filtration barrier ultrastructure, due to their shared functional, morphological and molecular features with vertebrate podocytes (Helmstadter et al., 2017; van de Leemput and Han, 2024; Zhang and Chen, 2014). While no model perfectly recapitulates human nephrotic syndrome, these models mimic certain aspects of podocyte damage and symptoms observed in patients, including the formation of glomerular segmental scars, podocyte foot process effacement, podocyte loss and albuminuria.

### 1.3 Podocytes maintain a highly organized actin cytoskeleton and interdigitating foot processes to form slit diaphragms, which are crucial for their filtration function

**FIGURE 2:**
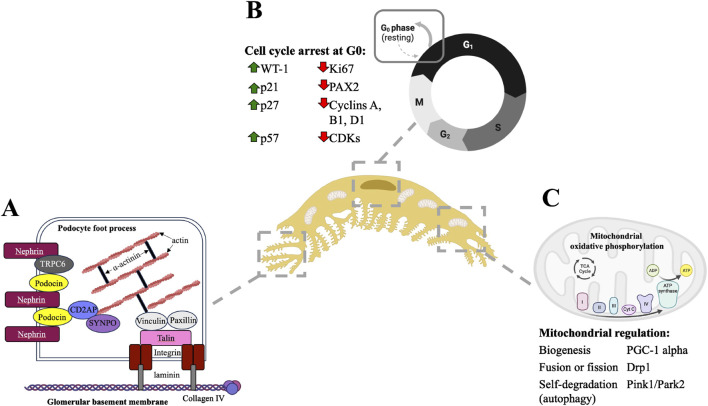
Podocytes are postmitotic cells comprised of a highly organized actin cytoskeleton, and these two cellular properties are supported by mitochondrial processes. **(A)** Podocyte foot processes are comprised of parallel actin filaments which are anchored to the cell membrane and to the underlying glomerular basement membrane by various actin-binding and actin-associated proteins **(A)** adapted from Lasagni et al., 2013; Curr Mol Med). **(B)** Podocytes undergo cell cycle arrest at the G0 resting phase. Within the nucleus, this terminally differentiated state is maintained through increased expression of cell cycle checkpoint proteins such as cyclin dependent kinase inhibitors and simultaneous decreased expression of proteins that promote cell cycle entry and proliferation. **(C)** The podocyte metabolic demands are largely due to the maintenance of their highly organized cellular structure and terminally differentiated state. Podocyte mitochondria have several adaptations to meet dynamic changes in metabolic demands, including biogenesis, fusion and fission to increase mitochondrial numbers and self-degradation through autophagy to remove damaged mitochondria and prevent excessive oxidative stress.

Podocytes are highly specialized, terminally differentiated visceral epithelial cells that have a complex cell structure comprised of a cell body, major processes and foot processes (Figure 1). Lasagni et al. (2013), Lennon et al. (2014), Faul et al. (2007) The foot processes are cellular extensions of podocytes that wrap around the outer surface of glomerular capillaries, forming interdigitations with foot processes of adjacent podocytes and creating filtration slits called the slit diaphragm. The slit diaphragm is a specialized cell-cell junction consisting of various structural proteins including nephrin, podocin and TRPC6, and plays a role in preventing the leak of plasma proteins into the urine (Wiggins et al., 2005; Fan et al., 2009; Gigante et al., 2009). The basal surfaces of foot processes are in direct contact with the glomerular basement membrane, comprised of extracellular matrix proteins, which underlie fenestrated endothelium (Figure 1). Lasagni et al. (2013), Lennon et al. (2014), Faul et al. (2007) At the molecular level, foot processes are comprised of a highly organized actin-based contractile apparatus, including an actin filament network, actin-associated proteins, membrane-associated proteins that anchor actin filaments to the cell membrane of the foot process, and membrane-associated proteins that anchor actin filaments to the glomerular basement membrane (Figure 2A). Lasagni et al. (2013), Lennon et al. (2014), Faul et al. (2007) RHO-family GTPases such as RHOA, RAC1, and CDC42 are known regulators of podocyte actin cytoskeletal dynamics by activating the formation of actin stress fibers (Mouawad et al., 2013; Matsuda et al., 2021; Boi et al., 2023). RHO GTPases respond to external stimuli, including mechanical stretch induced by changes in glomerular capillary blood flow, by cycling between an active GTP-bound and inactive GDP-bound conformation that activates or deactivates, respectively, their downstream effectors. This coordination results in modulating podocyte morphology, actin cytoskeleton dynamics, and slit diaphragm integrity, critical for maintaining glomerular filtration function (Mouawad et al., 2013; Matsuda et al., 2021; Boi et al., 2023).

### 1.4 Podocytes are terminally differentiated post-mitotic cells

The highly organized podocyte actin cytoskeleton architecture is associated with their terminally differentiated state (Figure 2B). Lasagni et al. (2013), Thomasova and Anders (2015), Liapis et al. (2013) Mature podocytes show quiescence and permanent arrest in the G0/resting phase of the cell cycle (Liapis et al., 2013). Similarly, other highly specialized cell types such as neurons and cardiomyocytes maintain a permanent exit from the cell cycle, coupled with a highly structured actin cytoskeleton (Thomasova and Anders, 2015). Since actin cytoskeleton dynamics and rearrangement underlie cellular changes including de-differentiation, migration and division, by nature any rearrangement in the podocyte actin cytoskeleton due to kidney injury or other external insults has potential to disrupt its core cell structure and function. Any attempt for podocyte self-renewal through the proliferation and replication would require significant actin cytoskeleton rearrangement, causing ultrastructural changes in their foot processes and could introduce ‘leaks’ in the glomerular filtration barrier. Under baseline conditions, mature podocytes are felt to have a very limited capability for cell division and poses a challenge for self-regeneration when lost through injury (Lasagni et al., 2013; Thomasova and Anders, 2015; Liapis et al., 2013). Podocytes gradually acquire their post-mitotic, terminally differentiated state as nephrogenesis progresses during kidney development. In the earlier stages, precursor cells that generate podocytes initially express high levels of proliferation markers such as Ki67, cyclins A, B1 and D1 and cyclin dependent kinases; while downregulating expression of cell cycle checkpoint proteins and cyclin dependent kinase inhibitors such as p21, p27 and p57 (Thomasova and Anders, 2015). Transcription factors like PAX2 that regulate mesenchymal-to-epithelial transition are also initially expressed at high levels but become downregulated as epithelial differentiation occurs (Quaggin, 2002; Cunanan et al., 2024). These cellular changes allow podocyte precursor cells to retain motility, facilitating cellular and morphological changes during development. At later stages of nephrogenesis, precursor cells begin to express markers of adult podocytes including Wilms Tumour-1 (WT-1) and podocalyxin, increase expression of cell cycle checkpoint proteins, and conversely downregulate cell proliferation proteins, leading to cell cycle exit and differentiation (Figure 2B). Thomasova and Anders (2015) The differentiation of podocyte progenitors are also coupled with the transition from a simple to highly organized actin cytoskeleton (Lasagni et al., 2013). Adult podocytes maintain a post-mitotic state through unique mechanisms: increased expression of cyclin-dependent kinase inhibitors (Combs et al., 1998; Shankland et al., 2000; Shankland et al., 2017; Hiromura et al., 2001; Barisoni et al., 2000; Cormack-Aboud et al., 2009), DNA regulatory mechanisms leading to cell cycle arrest (Shankland et al., 2017; Shankland et al., 1999), expression of podocyte histone deacetylases which modulate DNA damage responses (Medina et al., 2023), lack of ability to establish an effective mitotic spindle which is required to complete cytokinesis (Lasagni et al., 2010), and abnormal M-type cyclin activity during mitosis (Shankland et al., 2017; Marshall and Shankland, 2007).

### 1.5 Podocytes require energy to maintain actin cytoskeleton dynamics, the slit diaphragm and their differentiated state

Podocytes require energy to maintain actin cytoskeleton dynamics, the slit diaphragm, and their differentiated state. While studies have shown mitochondria contribute to podocyte energy metabolism, there are also reports that have shown podocytes primarily rely on anaerobic glycolysis instead of mitochondrial metabolism to maintain glomerular filtration (Brinkkoetter et al., 2019). Other studies have also established that podocytes are not as reliant on mitochondrial oxidative phosphorylation as other renal cells, such as proximal tubules, which have higher metabolic demands and are more susceptible to ischemic injury (Gobe et al., 1999; Wei et al., 2010). Nonetheless, transmission electron microscopy reveals a concentration of mitochondria within the podocyte cell body and foot processes, suggesting some involvement in cellular function and homeostasis (Imasawa and Rossignol, 2013; Abe et al., 2010). To adapt to changes in energy demand, podocyte mitochondria can undergo biogenesis, fusion or fission, mediated by proteins such as PGC-1 alpha and DRP1, to modulate mitochondrial dynamics as needed (Figure 2C). Additionally, podocytes maintain mitochondrial control through autophagy, a process mediated by proteins such as PINK1 and PARK2, which facilitates the degradation of damaged mitochondria to preserve cellular integrity (Handschin and Spiegelman, 2006; Fontecha-Barriuso et al., 2020; Elgass et al., 2013; Chen et al., 2020; Galluzzi et al., 2017; Jiang et al., 2020).

### 1.6 Podocyte loss results from disruptions in essential functions, including actin cytoskeleton integrity, slit diaphragm structure, cell differentiation and metabolism

#### 1.6.1 Actin cytoskeleton and slit diaphragm

Podocytes are continuously exposed to significant mechanical stress generated by the flow of glomerular ultrafiltrate (Faul et al., 2007; Blaine and Dylewski, 2020). Podocytes respond by modifying their foot process actin cytoskeleton. Expectedly, disruptions in actin cytoskeleton structure have been linked to podocyte loss in glomerular disease. One of the major causes of podocyte disruption are pathogenic variants in genes that encode actin binding and slit diaphragm proteins (Faul et al., 2007; Blaine and Dylewski, 2020). Some genes that contribute or regulate the actin cytoskeleton are *ACTN4*, *ARHGAP24* and *INF2,* pathogenic variants in which are causative for adult-onset FSGS (Akilesh et al., 2011; Schell and Huber, 2012; Brown et al., 2010; Sun et al., 2021; Kaplan et al., 2000). Mice deficient in or with a mutant form of alpha-actinin-4 developed podocyte damage and glomerular disease (Kos et al., 2003; Michaud et al., 2003; Yao et al., 2004). ARHGAP24 inhibits cell spreading and motility in podocytes by regulating RHOA/RAC1 signalling (Matsuda et al., 2021); consequently, *Arghap24* knockdown in mouse podocyte cell cultures demonstrated abnormally increased motility associated with actin cytoskeleton disruption (Akilesh et al., 2011; Schell and Huber, 2012). INF2 regulates podocyte actin dynamics and slit diaphragm protein trafficking by opposing RHO-mediated actin polymerization and rearrangement (Brown et al., 2010; Sun et al., 2013). As a result, kidney biopsy tissue obtained from a patient with a pathogenic variant in *INF2* showed abnormal aggregation of podocyte actin filaments associated with podocyte foot process effacement (Brown et al., 2010). Cultured human podocytes with INF2 knockdown showed a deregulated podocyte actin cytoskeleton and disrupted trafficking of slit diaphragm proteins (Sun et al., 2013). Variants in nephrin (*NPHS1*) and podocin (*NPHS2*), both encoding slit diaphragm transmembrane proteins, are also causative for Mendelian forms of nephrotic syndrome (Verma et al., 2018; Kestila et al., 1998; Ruotsalainen et al., 2000; Welsh and Saleem, 2010; Boute et al., 2000).

Experimentally induced podocyte injury through toxins or external insults also results in disrupted actin cytoskeleton organization. Exposure of podocytes to puromycin aminonucleoside causes altered intracellular localization of slit diaphragm proteins nephrin and podocin with disruptions in scaffolding protein CD2AP, resulting in decreased binding and depolymerization of actin filaments (Saleem et al., 2002). Adriamycin injured mice with knockout of synaptopodin (*Synpo*), which encodes an actin-associated protein, showed worsened podocyte injury and glomerular disease due to loss of actin stress fibers, impaired cell migration, mislocalization and decreased expression of alpha-actinin-4, (ACTN4) and altered activity levels of RHOA and RAC1 (Ning et al., 2020). In another model, exposure of podocytes to palmitic acid led to disruptions in actin cytoskeleton integrity, as well as decreased levels of actin-associated proteins including nephrin, vimentin, and the α3 chain of type IV collagen (CO4A3), overall culminating in cell loss (Martinez-Garcia et al., 2015). Furthermore, mouse podocytes grown in a high glucose environment led to abnormally increased activation of FYN/ROCK signalling, resulting in the phosphorylation of paxillin, interrupting the link between the foot process actin cytoskeleton and the glomerular basement membrane. These changes led to decreased levels of synaptopodin, marked actin cytoskeleton rearrangement and increased cell motility which exacerbated podocyte loss and glomerular dysfunction (Lv et al., 2016). HIV-1 protein NEF also promotes actin cytoskeleton changes in podocytes due to the ability of NEF to alter the activity levels of actin cytoskeleton regulators RHOA and RAC1 (Lu et al., 2008). These cellular and molecular effects led to podocyte damage, albuminuria and glomerular disease consistent with pathological findings in HIV-associated nephropathy (HIVAN) (Lu et al., 2008).

Taken together, these findings demonstrate that actin cytoskeleton integrity is crucial for healthy podocyte function. Regardless of underlying cause, alterations in normal podocyte actin cytoskeleton and foot process dynamics are strongly linked to overall loss (Faul et al., 2007; Blaine and Dylewski, 2020).

#### 1.6.2 Differentiated state and cell cycle abnormalities

**FIGURE 3:**
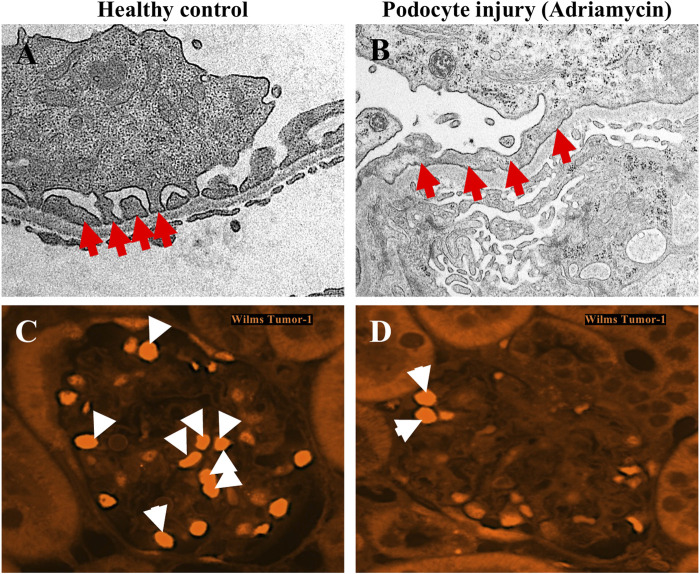
Podocyte foot process effacement and podocyte loss resulting from adriamycin injury. **(A,B)** Transmission electron microscopy images of podocytes from a healthy mouse glomerulus shows normal podocyte foot process morphology, characterized as interdigitating finger-like projections (red arrows) from the cytoplasm of podocytes which are in direct contact with the underlying glomerular basement membrane **(A)**. In mice with podocyte injury due to adriamycin, the characteristic interdigitating pattern of podocyte foot processes is lost (red arrows), and instead are observed as a continuous, flattened structure lining the glomerular basement membrane. **(C,D)** Immunofluorescence staining for podocyte nuclear marker Wilms Tumour-1 (WT-1) demonstrates decreased number of podocytes (white arrows) in adriamycin-injured mice (**D**) compared to healthy controls (**C**).

Podocyte cell cycle re-entry has been observed in human glomerular diseases such as IgA nephropathy, collapsing FSGS, HIVAN, lupus nephritis, and in experimental models of podocyte injury including adriamycin nephropathy (Figure 3), aminoglycoside-induced nephrosis, and TGF-beta overexpression in cultured mouse podocytes (Thomasova and Anders, 2015; Liapis et al., 2013; Barisoni et al., 2000; Barisoni et al., 2007). In these disease conditions, podocytes show a maladaptive attempt for cell cycle re-entry indicated by abnormal levels of cell cycle regulator proteins cyclins and cyclin-dependent kinase inhibitors, binucleation, micronuclei formation, and abnormally-formed mitotic spindles (Thomasova and Anders, 2015; Liapis et al., 2013; Barisoni et al., 2000; Barisoni et al., 2007). For example, in patient biopsies with HIVAN and collapsing FSGS, the loss of expression of cell cycle inhibitor proteins p27, p57 and cyclin D1, coupled with the abnormal re-expression of proliferation markers Ki67 and cyclin A, was observed specifically in podocytes that are found in areas of glomerular collapse (Barisoni et al., 2000). Increased levels of mitotic arrest deficient 2-like protein 2 (MAD2B), which regulates metaphase-to-anaphase transition, was also observed in FSGS patients and adriamycin treated mice and associated with podocyte cell cycle re-entry (Bao et al., 2021). These findings thus suggest that abnormal podocyte cell cycle re-entry plays a role in glomerular disease progression.

Under normal conditions, podocytes maintain cell quiescence but there are a number of pathologic or stress-inducing stimuli that can trigger them to undergo cell cycle re-entry including lupus, high glucose, ionizing radiation, fibroblast growth factors, and drugs that can interfere with cytoskeletal assembly stability (Kriz et al., 1995; Hagen et al., 2016; Vakifahmetoglu et al., 2008; Surova and Zhivotovsky, 2013; Xie et al., 2019). Under glomerular disease conditions characterized by podocyte loss, it is possible that the remaining podocytes attempt self-renewal to a certain extent (Cunanan et al., 2024). When attempting cell cycle re-entry, podocytes are forced to override certain checkpoints such as G2/M arrest (Lasagni et al., 2013; Thomasova and Anders, 2015; Liapis et al., 2013). This enables podocytes to undergo DNA synthesis and chromosome segregation, but the lack of sufficient actin dynamics renders them unable to complete cytokinesis efficiently. This generates podocytes with chromosomal irregularities and multinucleated cells that render them genetically unstable, leading to necrosis, apoptosis, podocyte detachment and loss, ultimately causing proteinuria and glomerulosclerosis, a phenomenon referred to as “mitotic catastrophe” (Lasagni et al., 2013; Thomasova and Anders, 2015; Liapis et al., 2013; Tian X. et al., 2023). In support of this, studies have found abnormal levels of binucleated podocytes in urine samples from patients with FSGS and IgA nephropathy (Hara et al., 2001; Lemley et al., 2002). In addition, mouse podocyte culture demonstrated binucleation and cell cycle re-entry upon stimulation with basic fibroblast growth factor. After puromycin aminonucleoside injury, the binucleated and proliferating podocytes were more susceptible to cell death compared to unstimulated podocytes (Hagen et al., 2016). As an alternative mechanism in response to pathological stimuli, podocytes can maintain a state of arrest in cell cycle checkpoints but instead attempt to undergo cell hypertrophy to compensate for podocyte loss (Lasagni et al., 2013; Thomasova and Anders, 2015; Liapis et al., 2013). In a case of human advanced diabetic glomerulosclerosis, most podocytes showed hypertrophy and partial detachment from the glomerular basement membrane (Liapis et al., 2013). In either case, podocyte cell cycle re-entry and growth arrest leading to hypertrophy both generate abnormal cells which ultimately cannot fully recapitulate the structure and function of normal podocytes (Lasagni et al., 2013; Thomasova and Anders, 2015; Liapis et al., 2013).

Overall, these findings demonstrate that podocytes retain a post-mitotic state through tightly controlled cellular and molecular mechanisms. Under conditions of significant stress, podocytes may be forced to undergo mitosis, but this results in nuclear irregularities that eventually lead to loss.

#### 1.6.3 Metabolism

Genetic abnormalities in key metabolic regulators, which lead to mitochondrial dysfunction and injury in podocytes, have been reported in both patients and rodent models of glomerular disease. Pathogenic variants in *COQ2* (coenzyme Q2) and *COQ6* (coenzyme Q6), which encode coenzymes required for the biosynthesis of the essential electron carrier ubiquinone in the mitochondrial transport chain, have been reported in familial FSGS (Diomedi-Camassei et al., 2007; Heeringa et al., 2011). Patients with *COQ2* mutations have increased numbers of dysmorphic mitochondria in podocytes, associated with decreased activities of electron transport chain complexes II and III and decreased ATP generation (Diomedi-Camassei et al., 2007). Podocytes also displayed severe foot process effacement and significant hypertrophy (Diomedi-Camassei et al., 2007). Furthermore, knockdown of *COQ6* in mouse podocyte cell cultures and zebrafish embryos resulted in apoptosis (Heeringa et al., 2011). Genetic defects in *PDSS2* (decaprenyl diphosphate synthase-2), encoding another enzyme involved in ubiquinone biosynthesis, were also found in patients with nephrotic syndrome (Lopez et al., 2006). Podocyte specific *Pdss2* knockout in mice resulted in foot process effacement and proteinuria (Peng et al., 2008). Podocyte-specific knockout of *Mtorc1* (mammalian target of rapamycin complex 1) in mice, which regulates mitochondrial activity and biogenesis, resulted in proteinuria and glomerular scarring (Godel et al., 2011). Similarly, podocyte-specific knockout of *Atg5* (autophagy related-5), a ubiquitin ligase involved in autophagy, resulted in increased oxidative stress, podocyte injury, glomerulosclerosis and proteinuria (Hartleben et al., 2010). Lastly, mice with a loss-of-function variant in *Crif1* (CR6-interacting factor-1), encoding a protein that participates in oxidative phosphorylation by regulating the insertion of polypeptides into the inner mitochondrial membrane, developed podocyte injury, glomerular sclerosis and marked albuminuria (Na et al., 2021). Studies using cultured mouse podocytes also showed loss of CRIF1 protein resulted in disrupted oxidative phosphorylation and abnormal F-actin aggregation, demonstrating that normal oxidative phosphorylation is necessary to maintain podocyte actin cytoskeleton and glomerular filtration barrier integrity (Na et al., 2021).

External insults can also lead to changes in expression levels of mitochondrial proteins and compromise function. For example, adriamycin-induced podocyte damage in rats, as well as in mouse podocyte cell cultures, resulted in decreased levels of PGC-1 alpha, a key regulator of mitochondrial biogenesis (Zhu et al., 2014). In these models, decreased PGC-1 alpha levels led to impaired oxidative phosphorylation, increased susceptibility to oxidative stress and apoptosis (Zhu et al., 2014). Similarly, mice with aldosterone-induced podocyte injury demonstrated increased renal oxidative stress and mitochondrial dysfunction, evident in reduced mitochondrial DNA copy number, mitochondrial membrane potential, and ATP production (Su et al., 2013). Proteinuria and podocyte injury were observed in these mice several days after the mitochondrial defects took place, demonstrating that mitochondrial dysfunction is an early event in podocyte injury (Su et al., 2013). Lastly, human podocytes exposed to a high glucose growth medium triggered an abnormal accumulation of pyruvate, the end-product of glycolysis (Feng et al., 2019). This resulted in interruption of downstream metabolic processes, oxidative phosphorylation and mitochondrial fragmentation leading to podocyte cell death (Feng et al., 2019).

Altogether, these findings demonstrate that podocytes have metabolic demands which are involved in maintaining podocyte structure and function. Mutations in key mitochondrial or metabolic regulators and various external insults can disrupt podocyte mitochondrial function and lead to decreased energy production, representing a mechanism that could contribute to podocyte loss.

### 1.7 Experimental approaches to mitigate podocyte loss

**FIGURE 4:**
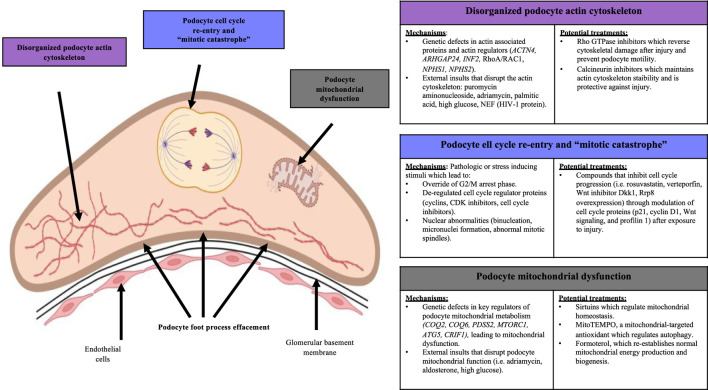
Mechanisms of podocyte loss and potential treatments. Disorganization of the podocyte actin cytoskeleton, cell cycle re-entry and mitotic catastrophe, and mitochondrial dysfunction are three cellular mechanisms that underlie podocyte loss. These mechanisms are also modifiable by potential treatments that can modulate the severity of podocyte injury.

An understanding of the mechanisms that maintain podocyte function and those that contribute to injury provide insights for therapeutic targeting in chronic kidney disease. Several studies have demonstrated that a variety of molecules and compounds can act on podocytes to stabilize their actin cytoskeleton, regulate homeostasis to maintain their post-mitotic state, and regulate mitochondrial dynamics and metabolism (Figure 4).

Mechanisms leading to podocyte actin cytoskeleton derangements can be reversed through certain drugs and molecules. Elevated levels of RHO GTPases and their regulators RHOA, RAC1 and ROCK contribute to podocyte dysfunction through disrupting the actin cytoskeleton resulting in abnormally increased motility (Akilesh et al., 2011; Babelova et al., 2013; Tian F. et al., 2023). These observations have led to the development of various agents that can inhibit RHO GTPases and their regulators. Mouse podocyte cultures exposed to mechanical strain simulating glomerular hypertension showed increased activity of RHOA/RAC1, actin cytoskeleton re-organization, de-differentiation of cells towards a mesenchymal phenotype, increased levels of fibrotic markers alpha-SMA (alpha-smooth muscle actin) and fibronectin, and decreased levels of podocyte proteins WT-1 and nephrin (Babelova et al., 2013). Inhibition of RAC1 and ROCK through EHT1846 and SAR407881, respectively, led to re-organization of the mouse podocyte actin cytoskeleton, reduction of mesenchymal de-differentiation, reduced fibrosis markers, and rescued WT-1 and nephrin levels similar to healthy controls (Babelova et al., 2013). Similar findings were reported in mice with experimental lupus nephritis where treatment with RHO GTPase inhibitor Fasudil prevented cytoskeletal breakage in podocytes (Tian F. et al., 2023), and in cultured human podocytes grown with damaging agents where glucocorticoid treatment markedly reduced RAC1 overactivity and cell motility (McCaffrey et al., 2017). Synaptopodin is also a regulator of RHO GTPases in podocytes. Dephosphorylation of synaptopodin by calcineurin, leading to synaptopodin degradation, was markedly increased in mice with lipopolysaccharide-induced podocyte injury (Faul et al., 2008). This also resulted in de-stabilization of the podocyte actin cytoskeleton. Cyclosporine A is a well-established treatment for FSGS and is known for its role in stabilizing the podocyte cytoskeleton (Cattran et al., 1999). Similarly, mice treated with cyclosporine A, a calcineurin inhibitor, prevented synaptopodin degradation and maintained actin cytoskeleton stability, demonstrating a protective effect against lipopolysaccharide injury (Faul et al., 2008). Mycophenolate Mofetil, an immunosuppressive drug, has been shown to stabilize the podocyte actin cytoskeleton in mice with podocyte injury from nephrotoxic serum. Mechanisms involved modulating calcium signaling, leading to reduced proteinuria and improved foot process architecture (Hackl et al., 2023). Altogether, these findings demonstrate that modulating podocyte actin cytoskeleton dynamics represents a therapeutic pathway.

Maladaptive podocyte mitosis can also be targeted to prevent podocyte loss. For example, the cell cycle protein p21 has a pro-survival effect in podocytes through modulating intrinsic DNA repair processes and inhibiting cell cycle (Cormack-Aboud et al., 2009). Mouse podocytes grown with rosuvastatin prior to induction of injury with puromycin aminonucleoside resulted in increased p21 levels and reduced apoptosis (Cormack-Aboud et al., 2009). Abnormally increased levels of YES-associated protein (YAP) was found in cultured mouse podocytes after adriamycin injury, accompanied by cell cycle re-entry and mesenchymal transition shown by increased cyclin D1 levels and downregulation of podocyte proteins (Xie et al., 2019). By contrast, treatment of podocytes with YAP inhibitor verteporfin prior to adriamycin injury resulted in decreased cell cycle re-entry and decreased mesenchymal transformation by reversing overexpression of cyclin D1 (Xie et al., 2019). Increased levels of telomerase reverse transcriptase (TERT) accompanied by abnormally increased activation of WNT signalling were observed in human and mouse kidneys with HIVAN (Shkreli et al., 2011). These were associated with cell cycle re-entry and loss of differentiated phenotypes, similar to immature podocyte progenitor cells. However, silencing TERT expression and treatment with WNT inhibitor DKK1 in mice reverted the podocytes to a differentiated state and reduced glomerular disease (Shkreli et al., 2011). Lastly, profilin 1 is a critical regulator of podocyte quiescence and its disrupted expression was accompanied by mitotic catastrophe leading to podocyte loss. Overexpression of ribosomal RNA processing 8 (RRP8) in podocyte cell cultures obtained from mice with profilin knockout partially reversed and prevented abnormal cell cycle re-entry (Tian X. et al., 2023). Thus, these studies demonstrate that targeting abnormal cell cycle re-entry in podocytes can re-establish podocyte quiescence and reduce the severity of glomerular injury.

Restoring podocyte mitochondrial homeostasis can also mitigate podocyte loss. Decreased levels of sirtuins have been associated with podocyte injury in rodents due to aging-associated podocyte loss (Liu T. et al., 2022; Chen et al., 2023). Sirtuins protect podocytes by deacetylating various mitochondrial targets including PGC-1 alpha, NF‐κB, FOXO, HIF-1 alpha, leading to the modulation of mitochondrial homeostasis. Compounds that increase sirtuin activity include natural products such as resveratrol, baicalin and grape seed extract, and synthetic compounds such as metformin, olmesartan, and SRT1720, all of which have been reported to reduce the severity of podocyte injury in experimental models (Liu T. et al., 2022; Chen et al., 2023). Decreased levels of PINK1/Parkin pathway-mediated mitochondrial autophagy was observed in Sprague-Dawley rats with experimental membranous glomerulonephritis, resulting in increased podocyte loss due to dysregulated mitochondrial function (Liu B. et al., 2022). This was reversed by treatment with MitoTEMPO, a mitochondria-targeted antioxidant, which increased PINK1/Parkin activity, resulting in modulated mitochondrial autophagy and reduced susceptibility to podocyte injury (Liu B. et al., 2022). Decreased levels of progranulin, a secreted glycoprotein that plays a role in mitochondrial autophagy, was associated with exacerbated mitochondrial damage and podocyte apoptosis in diabetic nephropathy (Zhou et al., 2019). *In vitro* treatment with progranulin on human podocytes cultured in high glucose medium attenuated the SIRT1-PCG-1 alpha/FOXI signaling pathway, resulting in re-establishment of mitochondrial membrane potential, improved mitochondrial biogenesis and decreased podocyte injury (Zhou et al., 2019). Similarly, placenta-derived mesenchymal stem cells were found to reduce the severity of high glucose induced podocyte injury by stimulating increased levels of mitochondrial autophagy mediated by PINK1/Parkin and the SIRT1-PCG-1 alpha pathways (Han et al., 2023). Lastly, human podocyte cultures and mice exposed to podocyte injury showed accelerated recovery when treated with formoterol, a β2-adrenergic receptor agonist (Arif et al., 2019). Formoterol was associated with increased PGC-1 levels, which resulted in re-establishment of normal mitochondrial structure, oxygen consumption rate, ATP generation, and enhanced podocyte mitochondrial biogenesis (Arif et al., 2019). Taken together, these studies demonstrate that targeting mitochondrial homeostasis in podocytes can attenuate injury and loss.

### 1.8 Future perspectives

Recent advances in single-cell transcriptomics, high-resolution imaging, and organoid models have significantly improved our understanding of podocyte structure and function in healthy and diseased states. Recent single-cell RNA sequencing (scRNA-seq) studies have identified key transcriptional programs and molecular signatures of podocyte injury in models of diabetic kidney disease, immune-mediated glomerulopathy, podocyte drug toxicities, and genetic podocytopathies (Table 1). Fu et al. (2016), Tsai et al. (2023), Chung et al. (2020), Bais et al. (2021) Gene ontology and pathway enrichment analyses reveal podocyte-specific biological and molecular processes that take place during podocyte injury and loss, all of which fall under the umbrella of dysregulated actin cytoskeleton, cell cycle, and metabolism. For example, in mouse models of diabetic kidney disease, pathway analysis of dysregulated genes revealed involvement in actin cytoskeleton organization, RNA processing and endoplasmic reticulum function, autophagy, altered cell survival, alterations in glucose and lipid metabolism, induction of apoptosis, and increased expression of extracellular matrix and matrix-modifying proteins in podocytes (Fu et al., 2016; Tsai et al., 2023; Chung et al., 2020). Furthermore, scRNA-seq has uncovered unique podocyte subpopulations with distinct responses to stress, suggesting heterogeneity in podocyte resilience and repair potential (Clark et al., 2022). As scRNA-seq datasets continue to grow, integrating transcriptomic findings with functional assays and spatial multi-omics will be essential to refine our understanding of podocyte pathology. Next, super resolution microscopy techniques including STED (stimulated emission depletion microscopy), STORM (stochastic optical reconstruction microscopy) and 3D-SIM (3 dimensional structured illumination microscopy) can reach resolutions down to 80–20 nm and have been used in obtaining nanoscale localization of podocyte slit diaphragm proteins and glomerular basement membrane proteins, nanoscopic resolution of actin cytoskeleton organization in podocytes under baseline and pathological conditions, and a higher resolution identification of foot process effacement in patient biopsies (Angelotti et al., 2021; Siegerist et al., 2018; Unnersjo-Jess, 2023). Atomic force microscopy has also been used to examine the mechanical properties of podocytes and the underlying glomerular basement membrane in healthy and diseased states (Siegerist et al., 2018; Wyss et al., 2011; Reynolds, 2020). Lastly, studies using glomerular organoids derived from human cells (i.e., induced pluripotent stem cells/iPSCs obtained from glomerular disease patients) have also provided accessible approaches to perform *in vitro* modelling of human podocyte injury and loss (Hale et al., 2018; Hale and Little, 2023; Lasse et al., 2023). These glomerular organoids have a more comparable morphology to human glomeruli *in vivo* and contain podocytes that recapitulate *in vivo* structure and apicobasal polarity, in comparison to cultured primary human and rodent podocytes. As a result, these glomerular models have helped advance the understanding of the podocyte transcriptome in patients with glomerular disease. Glomerular organoids have also served as valuable tools for drug screening to determine the efficacy of compounds that can mitigate abnormalities in podocyte structure and function (Hale et al., 2018; Hale and Little, 2023; Lasse et al., 2023).

**TABLE 1:** Summary of findings from single cell RNA sequencing studies, demonstrating abnormalities in the podocyte transcriptome under disease conditions.

Glomerular disease	Disease model	Pathways involved
Diabetic Nephropathy	Type I diabetes (streptozotocin injection with eNOS knockout)	Upregulated: cell death, actin organization
		Downregulated: RNA processing, endoplasmic reticulum (ER) function (mTOR signaling, autophagy, ER stress response)
	Type II diabetes (db/db leptin receptor-deficient mice)	Dysregulated: autophagy, cell survival, cell death
	Type II diabetes (ob/ob leptin-deficient mice)	Dysregulated: glucose and lipid metabolism
		Upregulated: apoptosis, matrix and matrix-modifying proteins
Lupus Nephritis/Goodpasture Disease	Nephrotoxic serum injection	Upregulated: cytoskeleton regulation, cell adhesion, inflammatory response, Hippo signaling, TGF-β signaling, NF-κB signaling, FoxO signaling
		Downregulated: podocyte differentiation
Podocyte Drug Toxicity	Adriamycin injection	Upregulated: p53 signaling
		Downregulated: podocyte markers
Podocyte-Specific Genetic Disease	CD2AP knockout	Upregulated: apoptosis, matrix proteins
	Doxycycline-induced CTCF knockout in podocytes	Dysregulated: mitochondrial function, cytoskeletal organization, cell-matrix adhesion
		Upregulated: *Col4a5* (potential cell-matrix adhesion)
		Downregulated: *Vegfa* (autocrine pro-survival ligand)

While the past 2 decades have largely expanded our knowledge of podocyte biology under health and disease, there are several withstanding challenges and gaps in knowledge in therapeutic development for podocyte loss. Despite promising pre-clinical data for a variety of compounds, there has been a lack of significant clinical translation (Meliambro et al., 2024). The current standard of care for podocytopathies largely rely on repurposed immunosuppressive treatments, with a relatively limited understanding of underlying mechanisms (Meliambro et al., 2024; Schonenberger et al., 2011; Cambier et al., 2018; Mirioglu et al., 2024). A major reason for the lack of therapeutic development for podocytopathies is the challenge of regenerating podocytes. In this review, we discussed mechanisms underlying the highly specialized, terminally differentiated nature of podocytes. These findings have led to the widely accepted notion that podocytes have a limited capacity for regeneration and loss is mostly irreversible. There is promise, however, with recent studies suggesting that there is a limited capacity for podocyte regeneration by other resident glomerular cell populations including parietal epithelial cells, which are proposed to have stem cell-like properties (Cunanan et al., 2024; Romagnani and Remuzzi, 2013; Ronconi et al., 2009; Lasagni et al., 2015; Wanner et al., 2014; Berger et al., 2014; Peired et al., 2014; Sagrinati et al., 2006; Kaverina et al., 2019), progenitor “renin lineage” cells (Pippin et al., 2013; Pippin et al., 2015; Eng et al., 2018), and bone marrow-derived mesenchymal stem cells (Zoja et al., 2012; Khalilpourfarshbafi et al., 2017; Wang et al., 2013). However, the concept of stem cell-based regeneration of podocytes from these cells is widely debated. Some studies demonstrate stem cell-like properties for potential podocyte progenitors (Romagnani and Remuzzi, 2013; Ronconi et al., 2009; Lasagni et al., 2015; Wanner et al., 2014; Berger et al., 2014; Peired et al., 2014; Sagrinati et al., 2006; Kaverina et al., 2019), while in contrast other studies support the contribution of these cells to pathogenic proliferation and the activation of pro-fibrotic pathways (Dijkman et al., 2005; Kuppe et al., 2019; Hakroush et al., 2014; Smeets et al., 2004; El Agha et al., 2017; Qin et al., 2023). These seemingly contrary studies have significant differences in their methodologies that could reconcile disparate results.

Another significant challenge in the treatment of glomerular diseases is the development of effective podocyte-directed drug delivery systems. Despite promising data from *in vitro* and animal studies showing therapeutic agents that can act on injured podocytes to re-establish their normal structure and function, our understanding of podocyte-targeted delivery systems is relatively lacking. For example, there are genes and signalling pathways, such as the Hippo/YAP signaling pathway and Tyro3, that are critical for podocyte survival but non-targeted delivery to other cell types can confer an oncogenic risk (Meliambro et al., 2024; Campbell et al., 2013; Zhong et al., 2023; Smart et al., 2018). Nanoparticle-based delivery systems can help reduce these off-target effects by protecting drugs from degradation and enabling controlled release. Nanoparticles can also be modified to carry ligands that specifically bind to podocyte receptors, such as nephrin and podocin, to improve podocyte delivery and uptake of drugs (Meliambro et al., 2024; Campbell et al., 2013; Zhong et al., 2023; Smart et al., 2018). Gene therapy, another promising approach, have been used to correct genetic mutations in podocytopathies (Mason et al., 2022; Daga et al., 2024; Ding et al., 2023). For example, adeno-associated virus (AAV) mediated delivery has been used in combination with CRISPR-Cas9 gene editing techniques to correct mutations in *COL4A3* and *COL4A5* in cultured podocytes from patients Alport syndrome, as well as to rescue *NPHS2* mutations in cultured podocytes from nephrotic syndrome patients (Mason et al., 2022; Daga et al., 2024; Ding et al., 2023). While nanoparticle and AAV-mediated drug delivery to injured podocytes show evidence reduced injury in these pre-clinical studies, both techniques also have significant challenges and limitations. Nanoparticles have instability issues such as aggregation, premature drug release, and degradation at inappropriate times, as well as difficulty in achieving consistent drug encapsulation, controlled release profiles, and proper biodistribution (Geszke-Moritz and Moritz, 2024). Gene therapy techniques such as AAV and CRISPR-Cas9 also have limitations, such as AAV’s limited packaging capacity (4.7 kb), immune responses to Cas9 proteins, and off-target effects that can cause unintended genetic alterations (Wang et al., 2024). While smaller Cas9 variants, dual AAV systems, and self-complementary AAV improve delivery and efficiency, they are limited by reduced cleavage activity, the need for simultaneous vector delivery, and higher risks at increased doses (Wang et al., 2024).

Overall, the translation of these findings from research to clinical practice require further rigorous investigation to determine patient safety and efficacy. Despite these withstanding challenges, our current knowledge of podocyte biology together with the identification of novel targets to attenuate podocyte injury, certainly provide promising insights to help overcome the lack of therapeutic development in this area.

## 2 Conclusion

The studies highlighted in this review demonstrate the challenge in therapeutic development for diseases of the podocyte, which are highly specialized in their morphology and function. The highly organized podocyte actin cytoskeleton is difficult to re-establish in the event of podocyte loss. Further, podocytes retain a quiescent, terminally differentiated cell state, making it unlikely for them to undergo cell division and self-renewal in the event of injury. Lastly, various insults leading to increased mitochondrial demands and stress also make podocytes vulnerable to injury. Overall, an understanding of the various cellular and molecular mechanisms that retain this intricate podocyte structure and function under healthy conditions, as well as the pathogenic mechanisms that lead to disruptions in the actin cytoskeleton, cell cycle arrest, and mitochondrial function has led to the identification of potential therapeutic targets that have the potential to mitigate the severity of glomerular diseases characterized by podocyte loss.
